# Supplementary material for: A Likelihood Approach for Real-Time Calibration of Stochastic Compartmental Epidemic Models
Source: PLoS Comput Biol. 2017 Jan 17;13(1):e1005257. doi: 10.1371/journal.pcbi.1005257 (PMC5240920; doi:10.1371/journal.pcbi.1005257)
Supplement: S1 File — (TAR.GZ) [file pcbi.1005257.s014.tar.gz › HSPH_Online-SI-Revision/PF08/n1-mild/n1-mild_PF2_table-8.pdf]

1	newly 3 weeks spec	14.	45.74	{3., 91.}	255.857	0.6164	newly 3 weeks cumul	37.	133.09	{13., 265.}	283.865	0.571515	newly 5 weeks spec	24.	39.41	{4., 80.}	113.542	0.479307	newly 5 weeks cumul	75.	215.3	{25., 406.}	215.467	0.525761	newly 63 weeks cumul	382.	460.46	{61., 781.}	59.0157	0.335263
2	newly 3 weeks spec	27.	28.49	{2., 62.}	65.8889	0.448694	newly 3 weeks cumul	86.	92.79	{10., 177.}	64.5465	0.389542	newly 5 weeks spec	28.	22.07	{1., 50.}	61.1071	0.556374	newly 5 weeks cumul	143.	139.92	{12., 301.}	59.4545	0.387182	newly 59 weeks cumul	317.	283.33	{33., 543.}	51.3218	0.377525
3	newly 3 weeks spec	15.	62.51	{6., 145.}	342.467	0.640968	newly 3 weeks cumul	45.	162.94	{14., 369.}	291.067	0.556635	newly 5 weeks spec	22.	60.81	{5., 128.}	207.773	0.590235	newly 5 weeks cumul	87.	285.22	{37., 606.}	254.828	0.573043	newly 68 weeks cumul	303.	631.63	{200., 868.}	124.729	0.47034
4	newly 3 weeks spec	11.	36.28	{1., 81.}	260.727	0.647738	newly 3 weeks cumul	28.	106.53	{5., 243.}	304.893	0.655137	newly 5 weeks spec	15.	34.51	{0., 77.}	174.467	0.632963	newly 5 weeks cumul	54.	176.31	{6., 356.}	255.13	0.640362	newly 62 weeks cumul	437.	407.86	{24., 776.}	52.5858	0.479264
5	newly 3 weeks spec	17.	50.81	{3., 105.}	228.765	0.552639	newly 3 weeks cumul	65.	146.07	{14., 305.}	159.431	0.478553	newly 5 weeks spec	31.	42.29	{3., 87.}	84.9355	0.452165	newly 5 weeks cumul	108.	235.65	{28., 461.}	151.287	0.478059	newly 64 weeks cumul	446.	473.33	{61., 803.}	48.8991	0.349708
6	newly 3 weeks spec	1.	62.16	{4., 151.}	6128.	1.56126	newly 3 weeks cumul	6.	166.67	{11., 426.}	2686.83	1.2048	newly 5 weeks spec	7.	56.16	{2., 119.}	723.143	0.898704	newly 5 weeks cumul	16.	280.68	{25., 621.}	1667.13	1.11427	newly 68 weeks cumul	409.	613.15	{176., 861.}	72.0513	0.406765
7	newly 3 weeks spec	31.	61.01	{7., 137.}	139.581	0.515601	newly 3 weeks cumul	65.	171.32	{14., 418.}	199.169	0.5278	newly 5 weeks spec	13.	53.37	{2., 102.}	337.923	0.700642	newly 5 weeks cumul	100.	282.85	{18., 622.}	211.37	0.558996	newly 67 weeks cumul	468.	600.96	{171., 884.}	56.3632	0.366699
8	newly 3 weeks spec	7.	44.04	{20., 77.}	529.429	0.743842	newly 3 weeks cumul	20.	130.89	{63., 230.}	554.45	0.764376	newly 5 weeks spec	14.	40.59	{14., 75.}	198.071	0.425795	newly 5 weeks cumul	41.	213.63	{104., 386.}	421.39	0.667973	newly 59 weeks cumul	476.	471.52	{202., 740.}	35.1891	0.17208
9	newly 3 weeks spec	17.	39.64	{14., 70.}	142.118	0.358258	newly 3 weeks cumul	37.	120.07	{50., 214.}	225.919	0.456771	newly 5 weeks spec	20.	34.38	{9., 57.}	92.2	0.299158	newly 5 weeks cumul	77.	191.1	{78., 322.}	152.208	0.357194	newly 58 weeks cumul	379.	401.74	{181., 678.}	43.1609	0.198014
10	newly 3 weeks spec	34.	40.46	{13., 77.}	57.7647	0.237085	newly 3 weeks cumul	83.	124.58	{42., 239.}	74.8193	0.239708	newly 5 weeks spec	34.	34.98	{10., 64.}	51.1176	0.283844	newly 5 weeks cumul	149.	197.22	{76., 362.}	62.8859	0.227214	newly 59 weeks cumul	381.	424.78	{140., 695.}	44.9134	0.210204
11	newly 3 weeks spec	5.	38.47	{8., 79.}	677.	0.8213	newly 3 weeks cumul	14.	114.58	{39., 237.}	720.	0.826183	newly 5 weeks spec	14.	31.21	{3., 60.}	147.214	0.463843	newly 5 weeks cumul	35.	180.86	{49., 368.}	422.514	0.646352	newly 58 weeks cumul	476.	375.12	{78., 650.}	42.2353	0.308268
12	newly 3 weeks spec	4.	37.6	{10., 80.}	843.	0.882703	newly 3 weeks cumul	11.	113.83	{40., 222.}	934.818	0.933875	newly 5 weeks spec	8.	33.56	{9., 61.}	333.25	0.639987	newly 5 weeks cumul	23.	184.27	{69., 360.}	701.609	0.820167	newly 59 weeks cumul	336.	430.47	{162., 707.}	50.4315	0.223631
13	newly 3 weeks spec	29.	29.45	{0., 63.}	68.2414	0.528541	newly 3 weeks cumul	92.	98.31	{5., 186.}	66.9239	0.454498	newly 5 weeks spec	28.	24.32	{0., 53.}	61.8571	0.539125	newly 5 weeks cumul	149.	150.13	{6., 285.}	63.604	0.456531	newly 60 weeks cumul	314.	299.01	{35., 567.}	54.6146	0.439794
14	newly 3 weeks spec	28.	47.15	{3., 104.}	117.893	0.507717	newly 3 weeks cumul	69.	135.89	{14., 290.}	138.159	0.453171	newly 5 weeks spec	32.	40.33	{4., 87.}	79.7813	0.447373	newly 5 weeks cumul	123.	220.78	{27., 453.}	120.667	0.436799	newly 64 weeks cumul	351.	455.82	{60., 794.}	65.3903	0.349913
15	newly 3 weeks spec	16.	49.26	{3., 104.}	237.75	0.575882	newly 3 weeks cumul	45.	142.93	{14., 297.}	243.8	0.544669	newly 5 weeks spec	24.	41.2	{2., 85.}	113.583	0.489416	newly 5 weeks cumul	89.	230.2	{24., 453.}	187.685	0.51168	newly 64 weeks cumul	454.	466.95	{55., 803.}	47.3414	0.353856
16	newly 3 weeks spec	21.	39.57	{3., 78.}	124.524	0.476564	newly 3 weeks cumul	39.	116.1	{7., 219.}	220.513	0.562289	newly 5 weeks spec	16.	37.86	{3., 80.}	169.875	0.526632	newly 5 weeks cumul	78.	192.95	{25., 355.}	174.218	0.531485	newly 62 weeks cumul	411.	446.75	{73., 796.}	54.3479	0.392339
17	newly 3 weeks spec	8.	43.61	{6., 97.}	463.375	0.716126	newly 3 weeks cumul	19.	119.26	{13., 259.}	544.842	0.735465	newly 5 weeks spec	11.	43.74	{3., 88.}	321.818	0.626874	newly 5 weeks cumul	37.	207.57	{26., 435.}	477.216	0.701936	newly 65 weeks cumul	358.	539.04	{102., 828.}	79.9106	0.3663
18	newly 3 weeks spec	10.	38.38	{13., 74.}	289.8	0.542958	newly 3 weeks cumul	30.	117.23	{44., 223.}	292.567	0.517848	newly 5 weeks spec	13.	31.88	{8., 57.}	162.615	0.436525	newly 5 weeks cumul	62.	185.39	{73., 355.}	203.79	0.423741	newly 58 weeks cumul	369.	402.66	{132., 659.}	44.0813	0.223535
19	newly 3 weeks spec	26.	44.32	{6., 97.}	117.615	0.48735	newly 3 weeks cumul	62.	119.67	{13., 259.}	133.726	0.46278	newly 5 weeks spec	40.	44.44	{3., 88.}	72.1	0.455314	newly 5 weeks cumul	140.	209.79	{26., 436.}	97.5071	0.428567	newly 65 weeks cumul	489.	539.87	{102., 828.}	48.3538	0.313887
20	newly 3 weeks spec	15.	35.54	{1., 81.}	174.8	0.57954	newly 3 weeks cumul	42.	106.01	{5., 244.}	184.929	0.558745	newly 5 weeks spec	12.	33.45	{0., 72.}	220.25	0.66526	newly 5 weeks cumul	79.	173.97	{6., 358.}	156.316	0.553545	newly 62 weeks cumul	337.	401.18	{24., 752.}	68.5519	0.48365
21	newly 3 weeks spec	3.	36.01	{2., 76.}	1119.	1.01993	newly 3 weeks cumul	24.	110.44	{9., 236.}	380.333	0.705864	newly 5 weeks spec	14.	33.6	{0., 72.}	189.714	0.671149	newly 5 weeks cumul	48.	178.67	{9., 356.}	299.063	0.681736	newly 62 weeks cumul	267.	380.09	{25., 736.}	91.4944	0.542404
22	newly 3 weeks spec	11.	47.92	{3., 104.}	359.636	0.680611	newly 3 weeks cumul	21.	139.21	{14., 278.}	576.905	0.739889	newly 5 weeks spec	8.	40.66	{4., 86.}	431.	0.703493	newly 5 weeks cumul	39.	224.26	{27., 435.}	489.692	0.698543	newly 64 weeks cumul	470.	466.08	{61., 783.}	44.2894	0.309485
23	newly 3 weeks spec	17.	38.69	{11., 79.}	141.235	0.347806	newly 3 weeks cumul	40.	120.03	{44., 219.}	202.975	0.416839	newly 5 weeks spec	12.	34.26	{8., 69.}	200.833	0.476859	newly 5 weeks cumul	68.	190.33	{70., 366.}	185.132	0.398734	newly 59 weeks cumul	373.	405.13	{175., 713.}	43.5094	0.199152
24	newly 3 weeks spec	11.	31.37	{2., 67.}	225.	0.615026	newly 3 weeks cumul	30.	97.08	{6., 202.}	254.6	0.566895	newly 5 weeks spec	19.	26.11	{2., 55.}	91.6316	0.495531	newly 5 weeks cumul	65.	151.76	{16., 297.}	170.277	0.492044	newly 61 weeks cumul	445.	356.17	{45., 661.}	44.8831	0.374271
25	newly 3 weeks spec	8.	39.89	{11., 79.}	401.375	0.619537	newly 3 weeks cumul	23.	120.42	{44., 227.}	424.	0.642529	newly 5 weeks spec	6.	32.01	{8., 60.}	443.167	0.712469	newly 5 weeks cumul	43.	188.92	{74., 315.}	341.628	0.574915	newly 58 weeks cumul	330.	395.54	{123., 665.}	50.8485	0.227532
26	newly 3 weeks spec	19.	33.23	{1., 66.}	123.105	0.557926	newly 3 weeks cumul	55.	103.	{5., 205.}	133.2	0.516454	newly 5 weeks spec	16.	27.55	{1., 57.}	114.188	0.505423	newly 5 weeks cumul	94.	161.52	{9., 314.}	117.128	0.478411	newly 61 weeks cumul	295.	373.09	{70., 639.}	63.3797	0.344782
27	newly 3 weeks spec	21.	36.05	{9., 69.}	95.	0.332926	newly 3 weeks cumul	42.	110.	{33., 217.}	172.286	0.397756	newly 5 weeks spec	24.	28.44	{3., 56.}	64.	0.4255	newly 5 weeks cumul	84.	171.02	{55., 338.}	119.81	0.33392	newly 58 weeks cumul	327.	355.83	{62., 625.}	49.8502	0.276088
28	newly 3 weeks spec	3.	46.29	{3., 99.}	1458.33	1.08297	newly 3 weeks cumul	9.	135.1	{14., 278.}	1406.44	1.02214	newly 5 weeks spec	4.	39.38	{4., 85.}	898.	0.899118	newly 5 weeks cumul	17.	217.7	{28., 433.}	1186.35	0.975141	newly 64 weeks cumul	356.	468.26	{61., 781.}	67.073	0.344317
29	newly 3 weeks spec	18.	31.09	{1., 67.}	123.833	0.560018	newly 3 weeks cumul	43.	97.33	{5., 201.}	167.558	0.526472	newly 5 weeks spec	35.	25.86	{1., 57.}	57.3714	0.549957	newly 5 weeks cumul	101.	151.4	{15., 297.}	99.7426	0.452896	newly 61 weeks cumul	354.	349.57	{37., 661.}	51.274	0.366167
30	newly 3 weeks spec	14.	42.12	{2., 95.}	231.	0.581943	newly 3 weeks cumul	49.	115.09	{12., 259.}	169.735	0.512448	newly 5 weeks spec	21.	42.4	{3., 85.}	144.762	0.520437	newly 5 weeks cumul	98.	200.95	{18., 424.}	141.827	0.483854	newly 65 weeks cumul	412.	524.24	{84., 825.}	63.432	0.375325
31	newly 3 weeks spec	26.	45.97	{3., 90.}	120.192	0.498776	newly 3 weeks cumul	72.	141.23	{18., 277.}	133.819	0.44517	newly 5 weeks spec	44.	37.41	{3., 76.}	56.4318	0.486778	newly 5 weeks cumul	142.	221.46	{31., 412.}	98.6479	0.414591	newly 63 weeks cumul	451.	444.88	{77., 757.}	45.9468	0.31887
32	newly 3 weeks spec	6.	30.38	{1., 67.}	439.333	0.755591	newly 3 weeks cumul	17.	93.86	{6., 184.}	472.353	0.683619	newly 5 weeks spec	8.	25.27	{0., 55.}	250.125	0.642072	newly 5 weeks cumul	37.	147.28	{16., 300.}	323.946	0.598215	newly 61 weeks cumul	420.	348.94	{28., 660.}	47.3905	0.406073
33	newly 3 weeks spec	16.	37.57	{2., 81.}	173.063	0.583769	newly 3 weeks cumul	37.	110.64	{5., 243.}	228.811	0.605583	newly 5 weeks spec	21.	35.13	{0., 77.}	119.	0.585706	newly 5 weeks cumul	77.	182.36	{6., 356.}	171.766	0.57816	newly 62 weeks cumul	388.	410.78	{19., 776.}	58.5567	0.47905
34	newly 3 weeks spec	7.	31.07	{1., 66.}	373.571	0.724789	newly 3 weeks cumul	27.	96.79	{5., 190.}	287.815	0.626684	newly 5 weeks spec	14.	26.5	{1., 57.}	130.286	0.516723	newly 5 weeks cumul	53.	152.61	{15., 297.}	219.151	0.559661	newly 61 weeks cumul	269.	352.73	{58., 639.}	72.7323	0.397183
35	newly 3 weeks spec	10.	45.93	{4., 91.}	383.1	0.691692	newly 3 weeks cumul	37.	132.99	{13., 263.}	283.541	0.573407	newly 5 weeks spec	22.	39.92	{4., 80.}	126.182	0.484201	newly 5 weeks cumul	79.	216.38	{25., 406.}	202.734	0.516779	newly 63 weeks cumul	462.	467.75	{61., 781.}	45.7208	0.316115
36	newly 3 weeks spec	18.	42.19	{2., 97.}	172.5	0.541755	newly 3 weeks cumul	39.	118.13	{12., 270.}	234.641	0.565717	newly 5 weeks spec	49.	40.88	{3., 85.}	58.7755	0.511262	newly 5 weeks cumul	114.	201.09	{17., 429.}	120.5	0.476089	newly 65 weeks cumul	404.	517.47	{78., 825.}	64.6312	0.385328
37	newly 3 weeks spec	12.	32.98	{1., 67.}	210.5	0.60925	newly 3 weeks cumul	26.	101.69	{5., 204.}	316.115	0.624508	newly 5 weeks spec	13.	27.31	{3., 57.}	145.462	0.510925	newly 5 weeks cumul	55.	159.44	{16., 305.}	218.436	0.536189	newly 61 weeks cumul	310.	358.64	{60., 639.}	59.8	0.349324
38	newly 3 weeks spec	35.	42.73	{6., 92.}	80.2	0.464671	newly 3 weeks cumul	65.	118.48	{13., 259.}	123.508	0.463189	newly 5 weeks spec	24.	41.51	{3., 85.}	118.958	0.493935	newly 5 weeks cumul	117.	202.8	{26., 421.}	114.393	0.453903	newly 65 weeks cumul	464.	517.01	{84., 825.}	50.4763	0.351032
39	newly 3 weeks spec	28.	46.11	{7., 85.}	100.75	0.429215	newly 3 weeks cumul	72.	144.73	{18., 263.}	132.236	0.43676	newly 5 weeks spec	31.	38.7	{4., 74.}	74.5161	0.436745	newly 5 weeks cumul	130.	227.61	{38., 409.}	107.238	0.405326	newly 63 weeks cumul	377.	462.13	{87., 743.}	55.8329	0.30548
40	newly 3 weeks spec	16.	48.99	{2., 104.}	239.063	0.611473	newly 3 weeks cumul	56.	132.35	{8., 302.}	176.411	0.523641	newly 5 weeks spec	15.	51.58	{2., 92.}	272.933	0.651286	newly 5 weeks cumul	83.	235.2	{17., 491.}	215.373	0.56526	newly 66 weeks cumul	397.	574.97	{80., 842.}	73.6549	0.403033
41	newly 3 weeks spec	13.	59.32	{1., 137.}	386.769	0.752546	newly 3 weeks cumul	28.	155.5	{4., 400.}	481.929	0.767152	newly 5 weeks spec	36.	54.21	{0., 106.}	101.139	0.614551	newly 5 weeks cumul	89.	268.17	{4., 609.}	235.18	0.66835	newly 67 weeks cumul	337.	583.47	{4., 884.}	103.967	0.578318
42	newly 3 weeks spec	5.	48.74	{2., 104.}	894.8	0.931556	newly 3 weeks cumul	20.	134.1	{8., 312.}	590.5	0.756253	newly 5 weeks spec	30.	50.51	{2., 92.}	110.767	0.504981	newly 5 weeks cumul	64.	234.59	{17., 491.}	292.703	0.618377	newly 66 weeks cumul	325.	567.41	{80., 842.}	101.283	0.450745
43	newly 3 weeks spec	39.	62.39	{5., 151.}	115.615	0.515373	newly 3 weeks cumul	61.	167.31	{11., 426.}	214.77	0.522974	newly 5 weeks spec	44.	56.33	{5., 119.}	84.7955	0.50413	newly 5 weeks cumul	146.	281.7	{27., 621.}	136.589	0.490922	newly 68 weeks cumul	494.	615.78	{181., 861.}	49.1984	0.325026
44	newly 3 weeks spec	19.	44.11	{15., 86.}	143.947	0.34844	newly 3 weeks cumul	73.	134.87	{48., 254.}	102.918	0.283188	newly 5 weeks spec	14.	38.66	{13., 75.}	185.714	0.431334	newly 5 weeks cumul	103.	214.96	{77., 401.}	120.252	0.308701	newly 60 weeks cumul	300.	472.21	{244., 769.}	68.9433	0.229089
45	newly 3 weeks spec	26.	35.37	{13., 67.}	62.1154	0.220262	newly 3 weeks cumul	61.	108.5	{44., 180.}	88.9508	0.249395	newly 5 weeks spec	23.	31.57	{11., 56.}	66.0435	0.256934	newly 5 weeks cumul	105.	172.88	{71., 314.}	78.6857	0.234672	newly 58 weeks cumul	383.	415.91	{187., 670.}	40.6031	0.18708
46	newly 3 weeks spec	13.	45.04	{6., 98.}	270.923	0.577115	newly 3 weeks cumul	26.	120.34	{14., 259.}	382.846	0.644324	newly 5 weeks spec	17.	45.04	{4., 88.}	198.471	0.538847	newly 5 weeks cumul	55.	212.06	{27., 436.}	306.218	0.589381	newly 65 weeks cumul	489.	536.16	{102., 828.}	49.0716	0.319892
47	newly 3 weeks spec	7.	41.44	{3., 85.}	512.571	0.777782	newly 3 weeks cumul	19.	123.49	{11., 249.}	567.211	0.754269	newly 5 weeks spec	6.	37.62	{3., 79.}	545.	0.733492	newly 5 weeks cumul	28.	201.15	{19., 401.}	633.464	0.791693	newly 63 weeks cumul	357.	441.79	{61., 781.}	64.7367	0.363475
48	newly 3 weeks spec	17.	37.84	{15., 70.}	129.529	0.316165	newly 3 weeks cumul	40.	113.31	{50., 206.}	183.825	0.398256	newly 5 weeks spec	17.	32.63	{9., 61.}	110.294	0.309774	newly 5 weeks cumul	72.	182.63	{73., 340.}	156.153	0.357314	newly 58 weeks cumul	326.	409.26	{142., 695.}	54.2638	0.218983
49	newly 3 weeks spec	2.	34.84	{1., 81.}	1662.	1.14354	newly 3 weeks cumul	12.	104.49	{5., 243.}	789.583	0.904046	newly 5 weeks spec	8.	32.26	{0., 66.}	340.75	0.756764	newly 5 weeks cumul	27.	170.78	{6., 356.}	554.889	0.820979	newly 62 weeks cumul	311.	392.28	{19., 748.}	73.7749	0.493867
50	newly 3 weeks spec	19.	43.09	{14., 81.}	137.	0.348826	newly 3 weeks cumul	64.	130.07	{48., 234.}	113.141	0.292323	newly 5 weeks spec	20.	35.54	{11., 68.}	95.	0.285992	newly 5 weeks cumul	105.	205.05	{72., 383.}	105.457	0.28273	newly 59 weeks cumul	406.	432.58	{179., 707.}	39.1232	0.183084
